# An Investigation of Factors Increasing the Risk of Aggressive Behavior among Schizophrenic Inpatients

**DOI:** 10.3389/fpsyt.2013.00097

**Published:** 2013-09-03

**Authors:** Michel Lejoyeux, Fabrizia Nivoli, Anne Basquin, Aymeric Petit, Florence Chalvin, Houcine Embouazza

**Affiliations:** ^1^Department of Psychiatry and Addictive Medicine, Bichat-Claude Bernard Hospital, AP-HP, Paris, France; ^2^Department of Psychiatry, Maison Blanche Hospital, Paris, France

**Keywords:** addiction, aggressive behavior, alcohol dependence, impulsivity, nicotine dependence, sensation seeking, schizophrenia

## Abstract

**Aim of the study:** This study tried to identify risk factors of aggressive behavior in a population of schizophrenic inpatients. We tested the association between aggressive behavior and socio-demographic characteristics, addictive disorders, history of suicide attempt, and sexual violence, impulsivity, and sensation seeking.

**Methods:** All consecutive schizophrenic inpatients (100) were assessed during 6 months. Aggressive behavior was quantified with a standardized scale, the Overt Aggression Scale (OAS). We studied socio-demographic characteristics and the history of suicide attempt and sexual violence with a specific standardized questionnaire. Addictive disorders were identified with the Fagerström and CAGE questionnaires and with the DSM-IV-R diagnostic criteria for nicotine, alcohol, cannabis opiates, and cocaine abuse and dependence disorders. Lastly, we studied sensation seeking with the Zuckerman scale and impulsivity with the Barratt scale.

**Results:** Linear regression identified four factors associated with aggressive behavior: male gender (odd ratio = 12.8), history of sexual violence (odd ratio = 3.6), Fagerström score (odd ratio = 1.3), number of cigarettes smoked each day (odd ratio = 1.16). Patients with nicotine use or dependence had significantly higher levels of OAS scores. This difference was not observed between patients with or without alcohol dependence. OAS scores were correlated to the number of cigarettes smoked each day and to Fagerström scores. Patients with a higher level of sensation seeking and impulsivity also had higher OAS scores.

**Conclusion:** A typical schizophrenic patient at risk of showing aggressive behavior is a man, who smokes and presents a history of sexual violence.

## Introduction

For professionals who treat addictive and psychiatric patients, aggressive behavior represents a risk to prevent (1). Aggression can be divided into affective and predatory violence. Affective violence involves hostile behavior as a reaction to some perceived threat, either from the environment or from an internal sense of fear. In contrast, predatory violence is planned, purposeful, and goal directed. The predator usually has no remorse and is comfortable using aggressive behavior to retaliate against others, gain a sense of control, or obtain a desired goal (2).

### Socio-demographic factors

The Epidemiologic Catchment Area study showed aggressive behavior in the general population being associated with younger age groups. Males perpetrated violent behavior 10 times more often than females (3). Two other factors associated with aggression are economic and intellectual levels. Aggressive behavior is three times as common among individuals with lower incomes and it is more frequent among subjects with mild mental retardation or lower intelligence. Among all predictive factors, a past history of aggression is the single best predictor of future violent behavior. Each prior episode of aggressive behavior increases the risk of future aggressive acts (4). Present and past sexual violence also increases the risk of aggressive behavior (5).

### Severity of schizophrenia

Among psychotic patients, Link et al. (6) reported that severity of delusion and hallucinations was significantly related to aggressive behavior (7). Schizophrenics are more likely to be violent if their hallucinations generate negative emotions (anger, anxiety, sadness) and if they have not developed successful strategies to cope with their voices (8, 9).

### Impulsivity and sensation seeking

A high level of sensation seeking is present in both dependent and aggressive patients (10). Aggressive behavior is often triggered by heavy drinking or drug intoxication. Other personality traits increasing the risk for violent behavior are impulsivity, low frustration tolerance, and the inability to tolerate criticism.

### Nicotine dependence and deprivation

Aggressive behavior is more frequent among nicotine dependents, especially when patients experiment nicotine deprivation during hospitalization (11). Parrott and Zeichner (12) assigned 35 male smokers to either a deprived or a non-deprived condition. A positive association between the urge for nicotine and aggression indexes was detected in the deprived and highly irritable group. Another study of schizophrenic patients (13) showed that one the principal symptom of nicotine withdrawal is agitation. Compared with non-smokers and smokers who received nicotine replacement, smokers without nicotine replacement had higher levels of irritability and agitation and were twice as likely to be discharged against medical advice. Moreover, a trend of higher rates of lorazepam use and a need for seclusion was observed among smokers without nicotine replacement therapy (11).

### Other addictive disorders

Drug and alcohol use disorders also increase the risk of violent behavior (4). At least half of all violent events, including murders, are preceded by alcohol consumption by the perpetrator of the crime, the victim, or both. Aggressive behavior associated with cocaine, crack, cannabis, or amphetamines differ by gender: men are likely to perpetrate aggression whereas women are more likely to be victims of aggression. Patients with addictive disorders are aggressive toward themselves and others. They commit suicide more impulsively and more violently (14).

### Aim of the study

In order to continue the risk factors analysis of aggressive behavior in schizophrenic patients, we studied a population of schizophrenic inpatients. We simultaneously assessed their level of aggressive behavior, impulsivity, sensation seeking, the presence and severity of addictive disorders and their history of suicide attempt, and sexual violence.

## Materials and Methods

The study was reviewed and approved by the ethical review board of the department of psychiatry and addictive medicine from Bichat (AP-HP) hospital. All patients, over 18 years old, participated on a voluntarily basis in the study and provided written informed consent. They were told they were participating in a clinical research. To ensure confidentiality, all identifying data were removed and all records were kept locked. We proposed the questionnaires to all consecutive schizophrenic patients admitted in the same department of psychiatry during the 6-months of the study. All patients fulfilled the DSM-IV-R diagnostic criteria of schizophrenia.

Patients were not pre-selected and they strictly reflect patients usually hospitalized. The department of psychiatry is located in a Parisian university hospital which serves the North of Paris (France). All patients were interviewed by a psychiatrist (Fabrizia Nivoli) or a psychologist (Anne Basquin) trained to use the study instruments. They were assessed during their first week of stay in the department. Assessment consisted in three sessions of an hour each of structured interviews. All patients could sustain their attention during the three sessions. All were abstinent from all substances during their hospitalization. Patients with alcohol dependence received benzodiazepines at admission in order to reduce alcohol withdrawal symptoms. The doses administered did not impair their cognitive state.

### Assessment of socio-demographic characteristics and aggressive behavior

We assessed aggressive behavior with a specific scale: a French version (15) of the Overt Aggression Scale (OAS) (16), which categorizes aggressive behavior according to four categories: verbal aggression, physical aggression against objects, physical aggression against self, and physical aggression against other people. Levels of severity can be identified within each category, with weighted scores ranging from 0 (lowest level of verbal aggression) to 6 (physical aggression against self or other people, resulting in serious injuries). The inter-rater reliability was 0.87 in previous studies for the total aggression score. In addition, patients filled a questionnaire specifically built for the study to assess their personal history of violent behavior, incarceration, arrests by the police, physical, or sexual aggression in childhood.

### Psychoactive agents use disorders

Cigarette smoking was studied with the Fagerström questionnaire (17). This test contains four yes-no and two multiple-choice questions. The average score in smokers is approximately 4–4.5. The DSM-IV-R criteria for nicotine dependence were also checked and we calculated the number of cigarettes smoked each day during the week before admission.

All patients answered the CAGE (Cut-Annoyed-Guilty-Eye Opener) questionnaire (18) to characterize their relation to alcohol. Item responses of the CAGE are scored 0 or 1. A total score of 2 or more is considered clinically significant. The quantity of drinks taken within a 24-h period during the last week was assessed with a specific questionnaire previously validated (19) – a drink being defined as the amount of alcohol (about 10 g) found in 300 ml of beer, 100 ml of wine, or 25 ml of whiskey. We also studied the number of days per week in which drinking occurred in the month before the interview and the number of acute alcohol intoxication. For cannabis consumption, we used a specific questionnaire and we quantified the number of joints smoked each day and the number of days of the week during which patients smoked cannabis. We also used the Cannabis Abuse Screening Test (20) and the Severity of Dependence Scale (21). The Cannabis Abuse Screening Test (11) basically designed for adolescents or young adults, identifies patterns of cannabis use leading to negative consequences on social adaptation and health. The scale has a sensitivity of 93% for cannabis use disorders. We finished this part of the assessment by checking the DSM-IV-R criteria for alcohol, nicotine, cannabis, opiates, and stimulants abuse and dependence.

### Impulsivity and sensation seeking

Patients filled in the Zuckerman sensation-seeking scale (22). Since all were French-speaking, a French translation of the Sensation-seeking scale (23) was given. This 72 item-scale gives five scores; F1: general factor, F2: thrill and adventure seeking, F3: experience seeking, F4: disinhibition, and F5: boredom susceptibility. The Barratt Impulsivity Rating Scale (24) gives four scores: total score, unplanned activity, cognitive impulsivity, and motor impulsivity.

### Analysis of data

For categorical variables (sex, consumption, or not of alcohol), we compared the level of aggressive behavior (OAS scale score) according to the presence or not of each parameter. For quantitative data (scores of impulsivity and sensation seeking, amount of alcohol, cigarettes, or cannabis), we made a correlation analysis between agitation scores and these variables. We applied a Bonferroni correction to control the increase in Type I error rate with multiple tests. We also performed a logistic regression to identify predictors of high OAS scores (superior to 3).

## Results

We proposed the questionnaires to 152 consecutive schizophrenic patients admitted in the same department of psychiatry. Fifty-two patients were not included in the study and 100 filled the questionnaires.

### Characteristics of patients not included

Among the 52 schizophrenic patients not included, 19 refused to participate, 7 did not understand the questionnaire because of cognitive deterioration, and 9 due to their lack of language comprehension. Sixteen patients were in a too severe psychiatric state to be interviewed. Their symptoms necessitated sedation and in nine cases physical contention. One quarter of the patients not included presented an alcohol or cannabis abuse or dependence disorder. Their mean age was 44 years (SD = 13.7) and 27 (52%) were women.

### Socio-demographic and clinical characteristics

The mean age of the patients included was 41.9 years (SD = 13.1, range = 18–84 years) (Table 1). The age was not correlated to OAS scores of agitation. Work status (employed or unemployed) was not associated with different levels of agitation. Patients hospitalized with or without their consent had an equivalent level of aggressive behavior. The group of patients with a history of sexual aggression in their childhood had a significantly higher level of aggressive behavior (mean OAS scores of 3.7 versus 1.9 in the group without history of sexual aggression, *p* = 0.004). Patients with a history of suicide attempts had higher OAS scores than those without (mean OAS scores of 3 versus 1.8 in the group without suicide attempt, *p* = 0.03). The number of previous suicide attempts was correlated to the OAS score (*p* = 0.001).

**Table 1:** **Comparison of agitation (OAS scores) according to. socio-demographic and clinical characteristics**.

	*N*	Mean OAS score	SD	Statistics
Men	42	1.78	2.4	Student *t* = −2.1, *df* = 98, *p* = 0.03
Women	58	2.9	3	
Brought in by the police	44	2.6	2.8	Student *t* = −0.6, *df* = 98, *p* = 0.5
Not brought in by the police	56	2.3	2.8	
Employed	33	2.7	3.1	Student *t* = −0.36, *df* = 98, *p* = 0.5
Unemployed	67	2.3	2.6	
History of incarceration	14	3	3.8	Student *t* = 0.73, *df* = 98, *p* = 0.46
No incarceration	86	2.2	2.6	
Hospitalization with consent	63	2.4	2.9	Student *t* = 0.03, *df* = 98, *p* = 0.95
Hospitalization without consent	37	2.4	2.7	
History of suicide attempt(s)	53	3	3	Student *t* = −2.1, *df* = 98, *p* = 0.03
No suicide attempt	47	1.8	2.4	
Hospitalization following agitation	41	2.2	2.6	Student *t* = −0.4, *df* = 98, *p* = 0.49
Not following agitation	59	2.6	2.9	
Physical violence in childhood	44	2.7	3	Student *t* = −0.44, *df* = 98, *p* = 0.44
No physical violence	56	2.2	2.6	
Sexual violence in childhood	30	3.7	3.2	Student *t* = −1.7, *df* = 98, *p* = 0.004
No sexual violence	70	1.9	2.4	

### Psychoactive substances consumption and addictive disorders

Patients with nicotine consumption had a higher level of aggressive behavior (mean OAS scores of 3.2 versus 2 in the group without nicotine) (Tables 2 and 3). Correlation studies of the OAS scores found a significant association with the number of cigarettes smoked each day, the number of days per month with cigarettes and the Fagerström score. No parameters related to alcohol consumption (number of drinks per day, number of drinking days per week) or alcohol use disorders (number of alcohol intoxications per week, mean CAGE scores) were correlated to the OAS score. Number of joints per day and number of days with cannabis were not correlated to the OAS score while scores of cannabis dependence (Severity of Dependence Scale, Cannabis Abuse Screening Test Score) were correlated to the OAS score. Only two patients presented opiates dependence and none presented stimulants dependence.

**Table 2:** **Comparison of agitation (OAS scores) according to the presence or not of psychoactive substances consumption**.

	*N*	Mean OAS score	SD	Statistics
Smoking nicotine	58	3	3.2	Student *t* = 2.4, *df* = 98, *p* = 0.01
No smoking	42	1.6	2	
Alcohol consumption	24	1.7	2.4	Student *t* = 1.3, *df* = 98, *p* = 0.17
No alcohol consumption	76	2.6	2.9	
Cannabis consumption	17	3.2	2.7	Student *t* = −1.2, *df* = 98, *p* = 0.23
No cannabis consumption	83	2.3	2.8	
Opiates consumption	2	2.8	2.6	Student *t* = −1.1, *df* = 98, *p* = 0.33
No opiate consumption	98	2.4	2.5	

**Table 3:** **Correlations between agitation (OAS score) and all quantitative parameters**.

	*R* (Pearson coefficient)	*p*
Age	−0.08	0.424
Number of arrests by the police	0.14	0.15
Number of days of incarceration	−0.06	0.5
Number of suicide attempts	0.31	0.001
Number of cigarettes/day	0.227	0.02
Number of days per month with cigarette	0.21	0.03
Fagerström score	0.23	0.01
Number of drinks/day	−0.07	0.46
Number of days/week with alcohol consumption	−0.11	0.25
Number of alcohol intoxication/week	0.009	0.93
Number of drinks/day	−0.07	0.46
Number of days/week with alcohol consumption	−0.11	0.25
CAGE score	0.09	0.35
Number of joints/day	−0.028	0.78
Number of days/week with cannabis	0.18	0.07
Severity of dependence scale score	0.27	0.007
Cannabis abuse screening test score	0.19	0.04
**BARRATT SCALE OF IMPULSIVITY**		
Total score	0.35	<0.001
Unplanned impulsivity	0.29	0.003
Motor impulsivity	0.29	0.003
Cognitive impulsivity	0.28	0.005
**ZUCKERMAN SCALE OF SENSATION SEEKING**		
General factor	0.18	0.06
Disinhibition	0.25	0.01
Danger and adventure seeking	0.004	0.9
Experience seeking	0.21	0.02
Boredom susceptibility	0.15	0.12

### Personality dimensions

The level of impulsivity assessed with the Barratt Scale was correlated to OAS scores (*p* < 0.001) (Table 3). A correlation was also found with OAS scores for all sub-scores of the Barratt Scale of impulsivity: unplanned impulsivity, motor impulsivity, cognitive impulsivity. Two sub-scores of the Zuckerman scale of sensation seeking – disinhibition and experience seeking – were significantly correlated to the OAS scores (*p* = 0.01 and 0.02, respectively) while the general factor of sensation seeking was not correlated.

### Logistic regression

In order to find an indication of the extent to which all these factors contribute to higher scores of OAS scale, we made a logistic regression (Table 4). We used as reference value, a score superior to 3 at the OAS scale. This statistical approach identified four factors associated to aggressive behavior: male gender (odd ratio = 12.8), history of sexual aggression (odd ratio = 3.6), Fagerström score (odd ratio = 1.3), number of cigarettes smoked each day (odd ratio = 1.18).

**Table 4:** **Multinomial logistic regression among patients presenting higher OAS (aggressive behavior) scores (Superior to 3)**.

Variable	Odd ratio	95% Confidence interval	Probability
Male gender	12.8	2.5–64	0.002
History of sexual violence	3.6	1.1–11.7	0.03
Cigarettes/day	1.16	1–1.3	0.02
Fagerström score	1.3	0.8–2	0.04
Cannabis score	1.16	1–1.3	0.12
DETA (alcohol) score	1.2	0.7–2	0.7
Cigarettes/day	1.1	0.9–1.2	0.2

*Reference category: patients with OAS scores >3, Likelihood ratio χ^2^ = 49.8, df = 12, p < 0.0001, 81% of the observations are classified by the model, sensitivity = 67%, specificity = 86%*.

## Discussion

### Socio-demographic characteristics

Like other studies, our work showed that the most important risk factor for violent behavior is gender. Male gender increases 12.8 times the risk of violent behavior. This result is consistent with the conclusions of a recent literature review (3) and with investigation who found that scores of aggression are significantly higher in male than in women among psychiatric inpatients. Another risk factor identified by our study is the history of sexual aggression. No other work, to our knowledge, identified this factor as associated with an increased level of violence. Many research (5) noted that victims of sexual violence are more exposed to engage themselves in violent behavior. This correlation has been demonstrated among young patients and alcohol or drug dependents. It had never been shown in schizophrenic inpatients. This first observation needs to be confirmed by further studies which could assess sexual aggression more precisely with the use of a more detailed questionnaire.

Other socio-demographic characteristics were not associated to violent behavior. Surprisingly, hospitalization following a state of agitation and/or police intervention had no OAS scores of aggressive behavior. These modalities of hospitalization are often considered by the medical team as risk factor of violence. Our data incite to minimize the weight of these factors and to give more importance to gender and the history of sexual aggression.

### Alcohol and nicotine dependence

Another result of our study shows that the level of nicotine consumption is more correlated to aggression than alcohol consumption. All parameters of nicotine consumption (number of cigarettes per day, number of days with cigarettes, Fagerström score) are correlated with OAS scores. None of the parameters related to alcohol consumption were significantly correlated to OAS scores. This difference between the impact of alcohol and nicotine on the risk of violent behavior had not been previously identified.

The lack of correlation between alcohol consumption and aggressive behavior differs from results of previous studies. MacArthur Foundation team (4) found that substance abuse and especially alcohol abuse increased the rates of aggression by up to five times. Their work however involved psychiatric patients who had been discharged from the hospital. Among schizophrenic patients still hospitalized, the effect of alcohol on the risk of aggressive behavior may be less significant since they do not drink during hospitalization. All our inpatients had stopped their alcohol consumption for at least 3 days. The hypothesis can be raised that the effect of alcohol on behavior is more immediate and related to current consumption. Alcohol increases aggressive behavior in the hours following its consumption (14) and its effects may disappear when patients have been weaned from alcohol.

Since our study is cross sectional, it does not allow us to give an explanation for the association between aggressive behavior and nicotine consumption. According to Allen et al. (13) who conducted a prospective study, most of schizophrenic patients who regularly smoke present a nicotine withdrawal syndrome during their hospitalization. Acute nicotine deprivation and withdrawal in smokers increases aggressive behavior and this effect is more pronounced in individuals with higher baseline irritability or hostility (13). Nicotine replacement and psychological intervention may reduce the agitation connected to nicotine withdrawal. None of our patients received a nicotine substitution during their hospitalization and all of them complained that they were not able to smoke as much as they wanted during their stay in hospital. They were authorized to smoke only a limited number of cigarettes. Nicotine withdrawal may thus explain at least part of their aggressive behavior. In daily practice, we recommend a systematic assessment of all addictive disorders and especially nicotine dependence during hospitalization in psychiatry. This assessment could prevent aggressive behavior related to intoxication or withdrawal. It could also represent the first step toward the recognition and the treatment of an addictive disorder too often undertreated and underdiagnosed.

Another explanation for the association between aggression and nicotine consumption is that patients with the most severe psychotic states are also those who smoke the most. Level of nicotine consumption may be considered as an indirect marker of psychiatric severity (13).

### Cannabis and other psychoactive substance use disorders

Concerning cannabis, we found a correlation of OAS scores with scores of dependence severity and not with the numbers of joints per day or the number of days with cannabis. The logistic regression integrating all potential risk factors for aggressive behavior did not identify cannabis dependence and/or consumption as significant. This observation differs from other studies which suggested that an increase in aggressive behavior may be explained by cannabis withdrawal symptoms (25). Another explanation is that cannabis dependence is associated with the most severe forms of psychiatric disorder whatever the initial diagnosis may be. No study however demonstrated a significant association between cannabis and aggressive behavior in a population of schizophrenics. Two patients presented opiates dependence and none stimulants consumption or dependence. This low rate of opiates and stimulants dependence can be explained by the fact that most of the addict patients are treated in a specialized department of our hospital.

### Sensation seeking and impulsivity

We found a correlation between OAS scores, sensation seeking, and impulsivity. Scores of impulsivity and sensation seeking did not appear in the logistic regression as risk factors for aggressive behavior. No other study found that sensation seeking is predictive of aggressive behavior in schizophrenic patients. Validity of the antisocial personality diagnosis in schizophrenic patients has been discussed (3).

### Limitations of the study

A first important limitation of this study is that one third of the patients (52 of 152) newly admitted in the department of psychiatry could not be included. These patients who refused to participate may correspond to the most severe cases of agitation. Due to this limitation, our study may only have concerned the less severely agitated patients. A second limitation is that we only studied schizophrenic patients. The majority of patients presenting opiates or stimulants dependence were not included since they are not hospitalized in the department of psychiatry. Our study due to its cross-sectional design only shows an association between clinical factors and levels of aggression. Only a prospective study could explain how these factors contribute to aggressive disorders in schizophrenics. Lastly, the concept of aggression has not yet been clearly defined with diagnostic criteria. Aggression does not appear in the glossary of the DSM. Bandura (26) already noted in 1973 that the study of aggression is like “entering a semantic jungle.”

## Conclusion

We did not identify a unique factor associated to aggression among schizophrenic patients. We found a constellation of socio-demographic and addictive characteristics. Aggressive behavior is more frequent in males, patients with a history of sexual abuse, and nicotine dependents. Among all addictive disorders, nicotine dependence was the sole addiction associated with an increased risk of aggression. The effect was not significant for alcohol, cannabis, and opiates dependence disorders. A systematic assessment of these factors could permit to anticipate clinical situations with a risk of aggressive behavior. The only “treatable” condition exposing to aggression could be the nicotine withdrawal syndrome in nicotine dependents. Nicotine replacement and psychological intervention on dependence may reduce the risk of aggressive behavior.

## Conflict of Interest Statement

The authors declare that the research was conducted in the absence of any commercial or financial relationships that could be construed as a potential conflict of interest.
